# Pathway-based expression profiling of benign prostatic hyperplasia and prostate cancer delineates an immunophilin molecule associated with cancer progression

**DOI:** 10.1038/s41598-017-10068-9

**Published:** 2017-08-29

**Authors:** Ankur Bhowal, Subhadipa Majumder, Subarna Ghosh, Sanmitra Basu, Debrup Sen, Susanta Roychowdhury, Sanghamitra Sengupta, Urmi Chatterji

**Affiliations:** 1 Department of Zoology, Cancer Research Laboratory, University of Calcutta, 35, Ballygunge Circular Road, Kolkata, 700019 West Bengal India; 2 Department of Biochemistry, University of Calcutta, 35, Ballygunge Circular Road, Kolkata, 700019 West Bengal India; 3 Molecular and Human Genetics Division, Indian Institute of Chemical Biology, 4, Raja SC Mullick Road, Kolkata, 700032 West Bengal India

## Abstract

Aberrant restoration of AR activity is linked with prostate tumor growth, therapeutic failures and development of castrate-resistant prostate cancer. Understanding the processes leading to AR-reactivation should provide the foundation for novel avenues of drug discovery. A differential gene expression study was conducted using biopsies from CaP and BPH patients to identify the components putatively responsible for reinstating AR activity in CaP. From the set of genes upregulated in CaP, FKBP52, an AR co-chaperone, was selected for further analysis. Expression of FKBP52 was positively correlated with that of c-Myc. The functional cross-talk between c-Myc and FKBP52 was established using c-Myc specific-siRNA to LNCaP cells that resulted in reduction of FKBP52. A non-canonical E-box sequence housing a putative c-Myc binding site was detected on the *FKBP4* promoter using *in silico* search. LNCaP cells transfected with the FKBP52 promoter cloned in pGL3 basic showed increased luciferase activity which declined considerably when the promoter-construct was co-transfected with c-Myc specific-siRNA. ChIP-PCR confirmed the binding of c-Myc with the conserved E-box located in the FKBP52 promoter. c-Myc downregulation concomitantly affected expression of FGF8. Since expression of FGF8 is controlled by AR, our study unveiled a novel functional axis between c-Myc, AR and FGF8 operating through FKBP52.

## Introduction

Androgens are known to be major stimulators of prostate tumor proliferation, as evident from initial arrest of metastatic growth by androgen ablation^1^. However, majority of patients eventually develop castration resistant prostate cancer (CRPC) which is typically androgen depletion resistant but androgen receptor (AR)-dependent^2^. In such cases, drugs that interfere with persistent intratumoral androgens provide an additional line of defense^3, 4^.

Androgen signaling is mediated by ARs which in its ligand unbound form interacts with multiple chaperones and co-chaperones to form a dynamic hetero-complex which allows the receptor to achieve the mature and functional conformation required for nuclear transportation and transcriptional activity^5^. Thus, drugs that would target these supplementary factors have emerged as an alternative or additional therapeutic support for the treatment of androgen-resistant aggressive prostate tumors^6, 7^. As a proof of concept, inhibitors of Hsp90, which is a molecular chaperone critical for maintaining proper folding of the AR, has been targeted in clinical trials for treatment of cancer^8^. Keeping in line, the immunophilins which act as AR co-chaperones, might also serve as promising therapeutic targets as they jointly control transactivation functions of AR without altering androgen binding^9, 10^.

The c-*Myc* proto-oncogene plays a crucial role in cell proliferation, differentiation, and apoptosis^11^. Consistent with the role of c-*Myc* in promoting cell proliferation, genetic alterations resulting in deregulation of c-*Myc* expression are common to a wide range of tumor types^12^. c-*Myc* over expression has been documented to promote development of prostate cancer^13^. c-*Myc* is up regulated both in androgen ligand-dependent prostate cancer and CRPC. Studies have confirmed that expressions of AR and c-*Myc* are strongly correlated in metastatic CRPC and c-*Myc* promotes the ligand-independent survival of the prostate cancer cell through its effect on AR^14^.

Benign prostatic hyperplasia (BPH) is another late onset proliferative condition of the prostate gland and often coexists with prostate cancer. BPH affects approximately 80–90% men in their 70 s and 80 s^15^. Although BPH is not a life threatening disease, it poses significant challenges on morbidity of the elderly population and is an economic burden. It is anticipated that the incidence of BPH will rise with increasing life expectancy in the elderly population of developing countries, like India. BPH basically involves stromal proliferation, which is another hallmark of growth of prostate tumor. Fibroblast growth factors (FGF) are multifunctional, heparin binding polypeptides that are shown to be expressed in BPH specimens^16^. Reports have stated a positive correlation between FGFs and expression of AR in prostate adenocarcinomas^17^. The development of androgen independence and progression towards metastasis constitute complex multifactorial events. In addition to abnormal AR signaling and c-Myc amplification, this involves multitude of growth factors and cytokines^18^. This warrants the need for a study which would address the functional connection of these factors with components of AR signaling.

The present study investigated differential expression of genes pertaining to the AR signaling pathway between BPH and prostate cancer patient samples, in anticipation that detection of specific genes and their associated components may provide the molecular cues underlying the mechanism of neoplastic development of the prostate gland and hold promises to eventually identify specific markers that demarcate disease states and progression.

## Results

### Expression of proliferation related genes in prostate cancer

**Figure 1:**
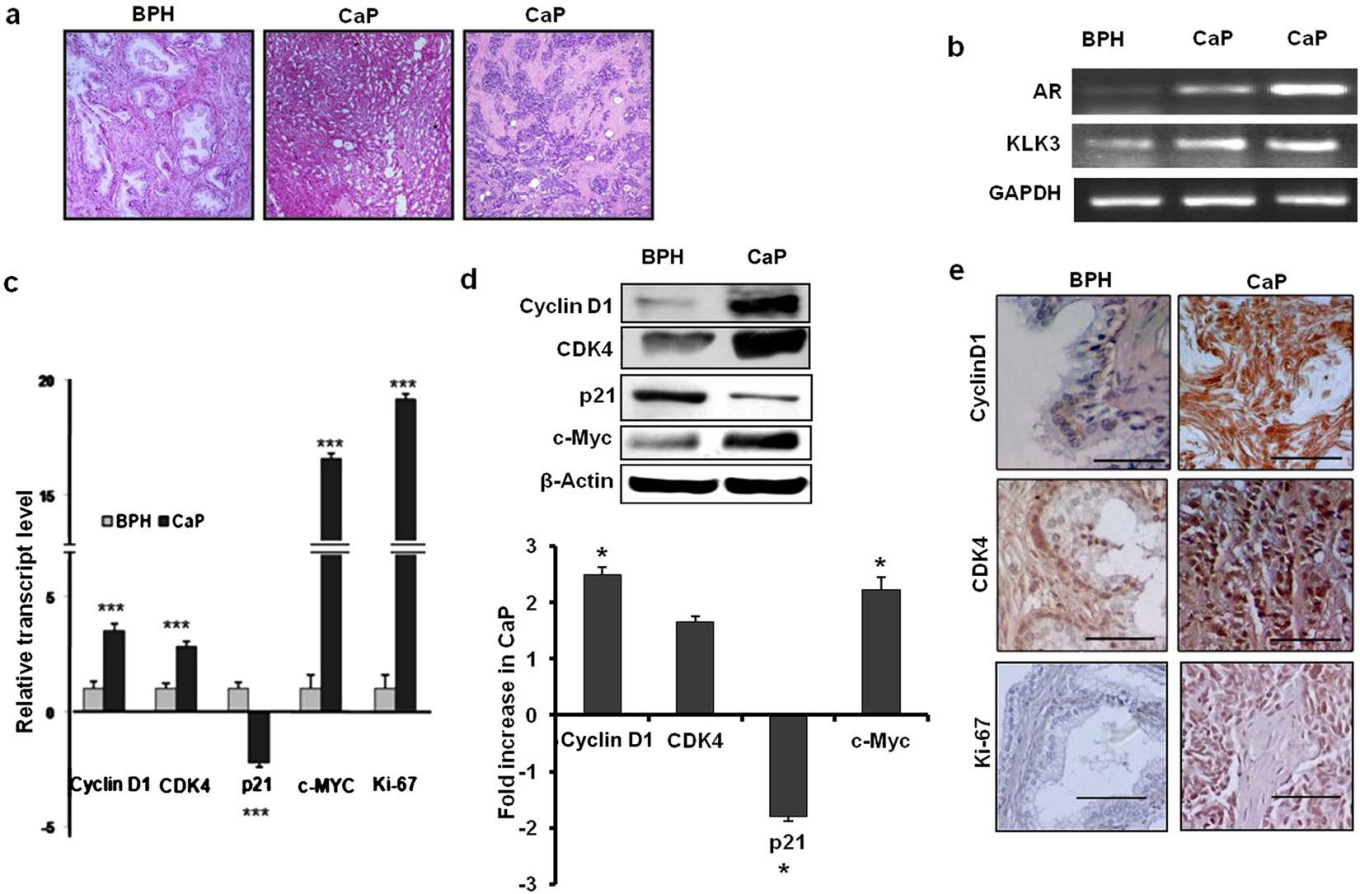
Histological and molecular criteria of BPH and CaP samples used in Microarray analysis. (**a**) H&E stained tissue sections of BPH and CaP samples. (**b**) Semi-quantitative RT-PCR based evaluation of AR and KLK3 genes in patient samples. Glucose-6-phosphate dehydrogenase (GAPDH) was used as an endogenous control. (**c**) Bar diagram representing relative expression of cell cycle and proliferation genes by quantitative RT-PCR. (***) indicated the P value < 0.001. 18 S rRNA gene was used as an endogenous control. (**d**) Representative western blots of cyclinD1, CDK4, p21 and c-Myc and in cancer and BPH samples. Human β-actin was used as loading control. Bar diagram in the lower panel showed the fold decrease of proteins in cancer tissues compared to BPH samples. (*) indicated the P value < 0.05. (**e**) Immunohistochemical evaluation of expression of cyclinD1, CDK4 and Ki-67 proteins in BPH and cancer samples (Bar: 50 µm).

In order to verify the authenticity of the prostate cancer and BPH tissues before the samples were subjected to microarray analyses, expression of several genes known to be functionally associated with androgen responsive pathway was examined using semi-quantitative and quantitative RT-PCR, western blot hybridization and immunohistochemistry. The genes selected for verification encode proteins namely *AR*, prostate specific antigen (*KLK3*), *cyclin D1*, *CDK4*, *p21*, *c-Myc* and *Ki-67*. The histological architecture of three specimens under study confirmed the status of the tissues. The tissue sections representing BPH sample showed hyperplasia of glandular tissue with infoldings within the lumen and intervening stroma. Sections of adenocarcinoma samples displayed cribriform glands or only sheets of cells with hyperchromatic nuclei without prominent lumen (Fig. 1a). Semi quantitative PCR confirmed higher expressions of *AR* and *KLK3* genes in tumor tissues compared to that in the BPH sample, which was considered as a reference control in the microarray experiment (Fig. 1b). The relative expression of *cyclin D1*, *CDK4* and *c-Myc* genes averaged for two cancer tissues indicated significant up regulation of all three cell cycle markers both at the transcript level (3.54, p = 0.0001; 2.85, p = 0.001; and 16.56 fold, p = 0.0001 respectively) and protein level (2.5, 1.66 and 1.8 fold respectively) (Fig. 1c and d). Immunohistochemistry confirmed a higher expression for cyclin D1 and CDK4 proteins in cancer samples (Fig. 1e). Both quantitative RT-PCR (19.2 fold, p = 0.0001) and immunohistochemistry showed a remarkably higher expression of Ki-67 in cancer tissues (Fig. 1c and d). On the other hand, p21 was down regulated in the cancer samples compared to the BPH control both at transcript (2.23 fold, p = 0.001) and protein (1.5 fold, p = 0.001) levels. Taken together these data indicated that prostate cancer tissues under study were indeed in a highly proliferative state and engaged actively in AR signaling pathway.

### Gene expression analysis of AR pathway

**Figure 2:**
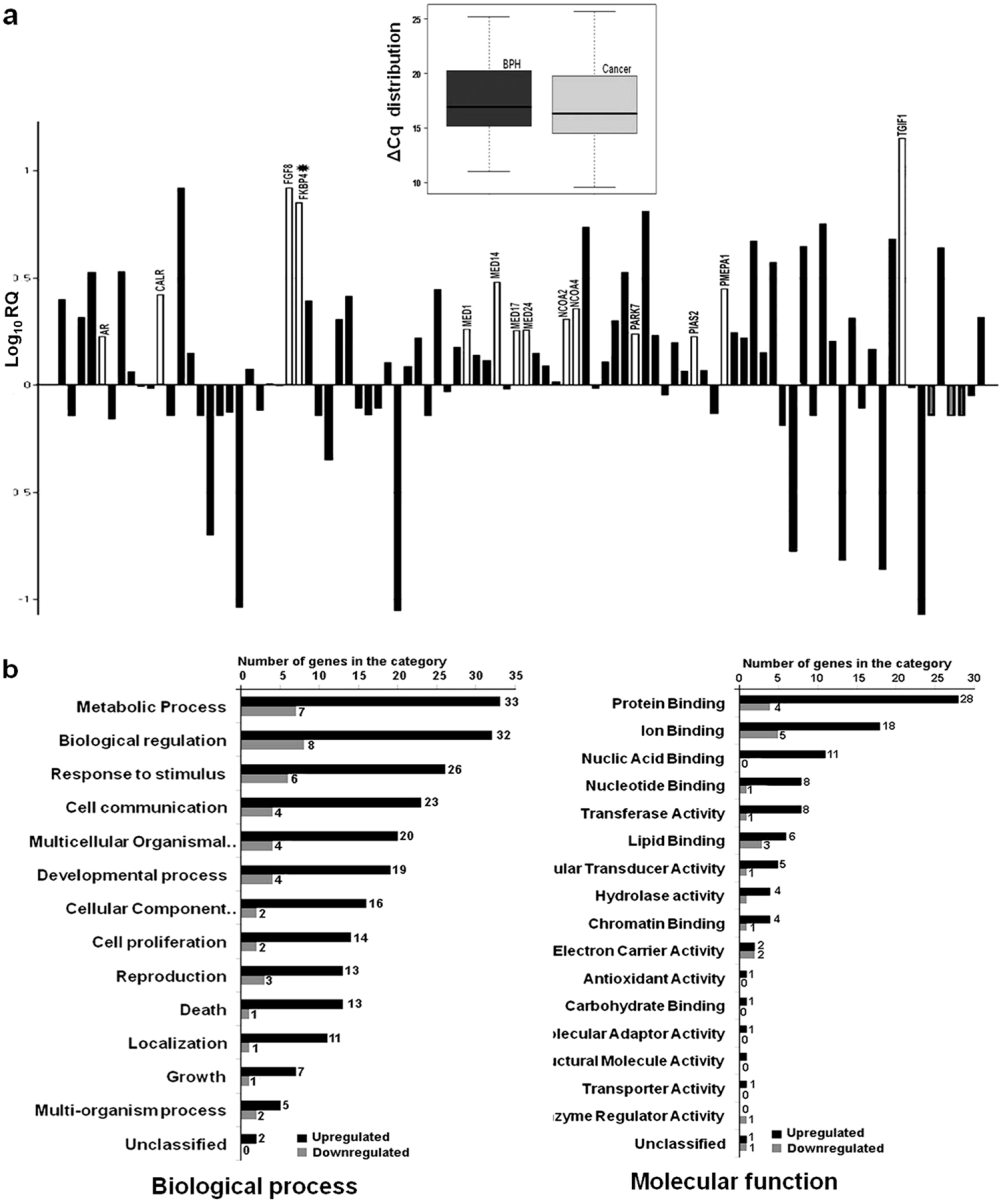
Relative expression and functional categorization of differentially regulated genes belonging to the AR receptor signaling pathway. (**a**) Bar diagram showing average fold difference of 94 genes in CaP with respect to BPH. The height of each bar represents Log_10_RQ of the genes. White colored bars represent over expressed genes belonging to the androgen/intracellular steroid receptor pathway. Box plot in the inset represents an overall higher expression of AR pathway genes in cancer. The bottom line, middle line, and top line of each box represent 25th percentile, median, and 75th percentile, respectively. Bars extend to the lowest value and to the highest value of ΔCq of each group. (**b**) Differentially regulated genes were categorized based on their affiliations to different Biological Processes or Molecular Functions. Each category was represented by two bars for genes up regulated and down regulated. The height of the bar indicates the number of input genes observed within the category.

The differences in gene expression between one BPH and two CaP tissues were analyzed by a pathway-based microarray analysis for 94 genes associated with AR signaling. BPH was used as the reference sample and a *p* value of ≤ 0.01 was considered as detection criteria with respect to 18 S rRNA and GAPDH, both as endogenous controls for differential expression. The assay revealed an overall up regulation of AR signaling pathway genes (Fig. 2a, inset). A total of 52 genes showed significant differences (more than 1.5 fold, *p*-value ≤ 0.01), with 28 genes showing more than 2-fold up regulation and 8 genes more than 2-fold down regulation in CaP (Fig. 2a). Regulation of metabolic process, biological regulation and response to stimuli were the three top-rank biological processes that were enriched (Fig. 2b). Among the genes which were significantly upregulated in prostate cancer were those related to protein binding and achieved the highest ranking in the molecular function category (Fig. 2b). Based on their co-occurrence in the Gene Ontology (GO) classification, a set of candidate genes were subjected to further molecular analyses (Table 1).

**Table 1:** Enriched GO categories and genes overexpressed in prostate cancer (>1.5 fold).

Sub-root	Category name and corresponding GO ID	Detectors (Genes)	rawP	adjP
Biological process	positive regulation of metabolic process ID:GO:0009893 ID:GO:0031325	SOAT1,RCHY1, POR, FOXO3, SRY, CALR, HPRT1, NCOA4, IL6, PIAS2,MED24, **AR**, MED17,SCRAB1, MED14, SPDEF, **FGF8**, MED1,NCOA2	4.49e-08 1.31e-07	5.47e-06 1.20e-05
Biological process	positive regulation of biosynthetic process ID:GO:0009891 ID:GO:0031328	SOAT1, POR, FOXO3, SRY, CALR, NCOA4, IL6, PIAS2, MED24, **AR**, MED17, SPDEF, MED14, SCRAB1,MED1,NCOA2	2.33e-08 1.80e-08	3.41e-06 3.29e-06
Biological process	intracellular steroid hormone receptor signaling pathway ID:GO:0030518 ID:GO:0030522 ID:GO:0030521	CALR, NCOA4, PEMPA1, PIAS2, **AR**, MED24, MED17, MED1, **FKBP4**, PARK7, NCOA2	1.23e-17 1.40e-14 2.95e-18	4.50e-15 3.41e-12 2.95e-18
Biological process	sex differentiation ID:GO:0007548	NCOA4, **AR**, MED1, **FKBP4**, **FGF8**, FOXO3, SRD5A1, SRY	2.21e-07	1.62e-05
Molecular function	transcription factor binding transcription factor activity ID:GO:0000989 ID:GO:0003713	MED24, MED17, TGIF1, MED14, NCOA4, MED1, NCOA2, PIAS2	1.93e-05 3.83e-06	P = 0.0002 4.47e-05
Molecular function	steroid hormone/ androgen receptor binding ID:GO:0005102 ID:GO:0051427 ID:GO:0035257	CALR, NCOA4, IL6, PIAS2, **AR**, RCHY1, MED24, MED17, MED14, MED1, **FKBP4**, **FGF8**, NCOA2	3.77e-06 2.79e-12 7.90e-13	4.47e-05 1.46e-10 8.29e- 11
Molecular function	vitamin D receptor & thyroid hormone binding ID:GO:0042809 ID:GO:0046966	MED24, MED17, MED14, MED1	7.55e-08 8.50e-07	1.59e-06 1.49e-05

*Similar biological processes and molecular functions were clubbed and presented in the same row

**The genes marked in bold were subjected to further molecular analyses.

### FKBP52, the immunophilin molecule, significantly up regulated in prostate cancer tissues

**Figure 3:**
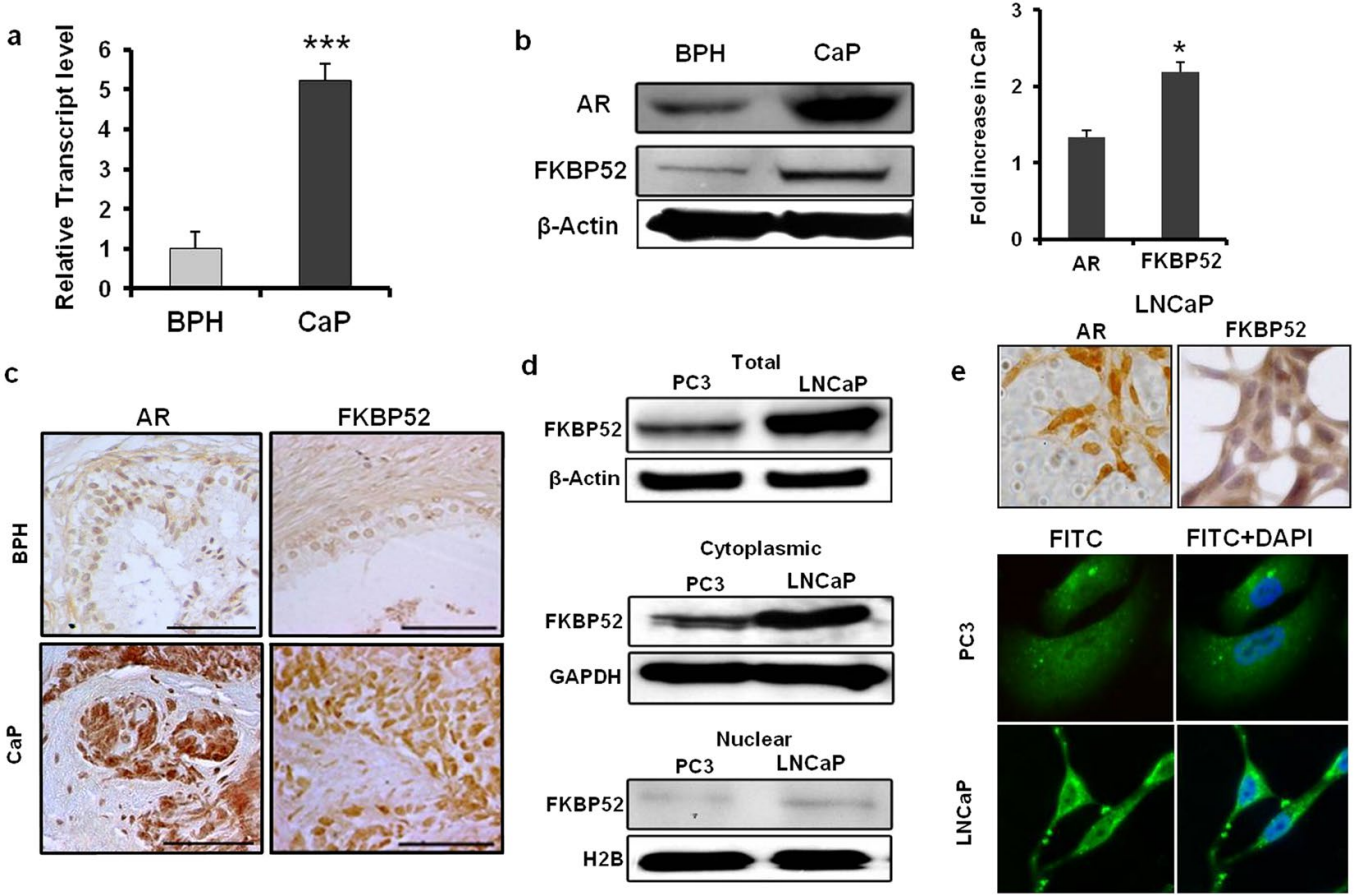
Expression of FKBP4 in tissues and human prostate cancer cell lines. (**a**) Column plot representing the relative transcript level of FKBP4 gene. FKBP4 was over expressed (P = 0.001) in cancer samples (n = 15) compared to BPH tissues (n = 15). 18 S rRNA gene was used as an endogenous control. (**b**) Western blots of AR and FKBP4 proteins in representative samples. Human β-actin gene was used as loading control. Histograms showed relative fold increase of protein in cancer samples (n = 6) with respect to BPH (n = 6) samples. Relative intensity of the band was normalized with respect to β-actin expression. (**c**) Immunohistochemical evaluation of expression of AR and FKBP52 proteins in CaP and BPH samples. Scale Bar: 50 µm. (**d**) Western blot analyses showing the distribution of FKBP52 in total, cytoplasmic and nuclear fractions from PC3 and LNCaP cell lines. β-Actin, GAPDH and H2B were used as endogenous controls for total, cytoplasmic and nuclear fractions, respectively. (**e**) Relative distribution of AR and FKBP52 in LNCaP cells by confocal microscopy. DAPI was used for nuclear staining. Magnification: 400x.

The pattern of differential expression of genes related to the AR pathway with >2-fold change in either direction was summarized in Table 2. The genes which were up regulated >5-fold in CaP included the immunophilin molecule, FK506 Binding Protein 4 (*FKBP4*; the protein encoded by this gene is known as FKBP52), fibroblast growth factor 8 (*FGF8*), claudin 3 (*CLDN3*) and transglutaminase 4 (*TGM4*). Since immunophilin molecules are known to directly regulate the AR signaling, we compared the levels of FKBP52 expression in a larger cohort of BPH (n = 15) and CaP (n = 15) samples. The relative expression of FKBP52 was upregulated by 5-fold at the transcript level and 2.2-fold at the protein level (p < 0.001) in CaP samples compared to BPH tissues (Fig. 3a and b). *FKBP4* gene was consistently over expressed in CaP tissues as the ΔCq estimates in 14 out of 15 CaP samples of *FKBP4* gene elevated by a range of 1.2–26.72 fold compared to the mean level of normalized *FKBP4* expression estimated in 15 BPH samples. Immunohistograms also supported higher expression of FKBP52 in CaP and that expression of FKBP52 correlated with the expression of AR in the human tissues (Fig. 3c). A similar result was observed in AR-responsive LNCaP cells, which showed higher FKBP52 expression in both cytoplasmic and nuclear fractions compared to that in the AR-unresponsive PC3 cells (Fig. 3d). Hence, all future studies were carried out with LNCaP cells. The expression of FKBP52 was higher in the cytoplasmic rather than the nuclear fraction in both cell types (Fig. 3e).

**Table 2:** Differentially regulated genes in prostate cancer with >3-fold changes in array plate.

Gene symbol	Gene name	Fold change
TGM4	transglutaminase 4 (prostate)	14.20
FGF8	fibroblast growth factor 8 (androgen-induced)	8.31
CLDN3	claudin 3	8.29
PART1	prostate androgen-regulated transcript 1	8.06
FKBP4	FK506 binding protein 4, 59 kDa	7.11
SPDEF	SAM pointed domain containing ets transcription factor	5.68
NKX3	NK3 homeobox 1	5.45
TGIF1	TGFB-induced factor homeobox 1	4.79
RDH11	retinol dehydrogenase 11 (all-trans/9-cis/11-cis)	4.70
SOAT1	sterol O-acyltransferase (acyl-Coenzyme A: cholesterol acyltransferase) 1	4.44
UGT2B15	UDP glucuronosyltransferase 2 family, polypeptide B15	4.38
SCARB1	scavenger receptor class B, member 1	3.74
BMX	BMX non-receptor tyrosine kinase	3.37
APPL1	adaptor protein, phosphotyrosine interaction, PH domain & leucine zipper containing 1	3.37
PAK6	p21(CDKN1A)-activated kinase 6	3.37
MED14	mediator complex subunit 14	3.02
UGT1A8	UDP glucuronosyltransferase 1 family, polypeptide A8	−11.95
HSD3B1	hydroxy-delta-5-steroid dehydrogenase, 3 beta- and steroid delta-isomerase 1	−11.27
CYP21A2	cytochrome P450, family 21, subfamily A, polypeptide 2	−11.27
TGFB1I1	transforming growth factor beta 1 induced transcript 1	−7.25
SRD5A2	steroid-5-alpha-reductase, alpha polypeptide 2 (3-oxo-5 alpha-steroid delta 4-dehydrogenase alpha 2)	−6.57
SHH	sonic hedgehog homolog (Drosophila)	−5.98
CYP11A1	cytochrome P450, family 11, subfamily A, polypeptide 1	−5.01

### FKBP4 promoter activity was controlled by c-Myc

**Figure 4:**
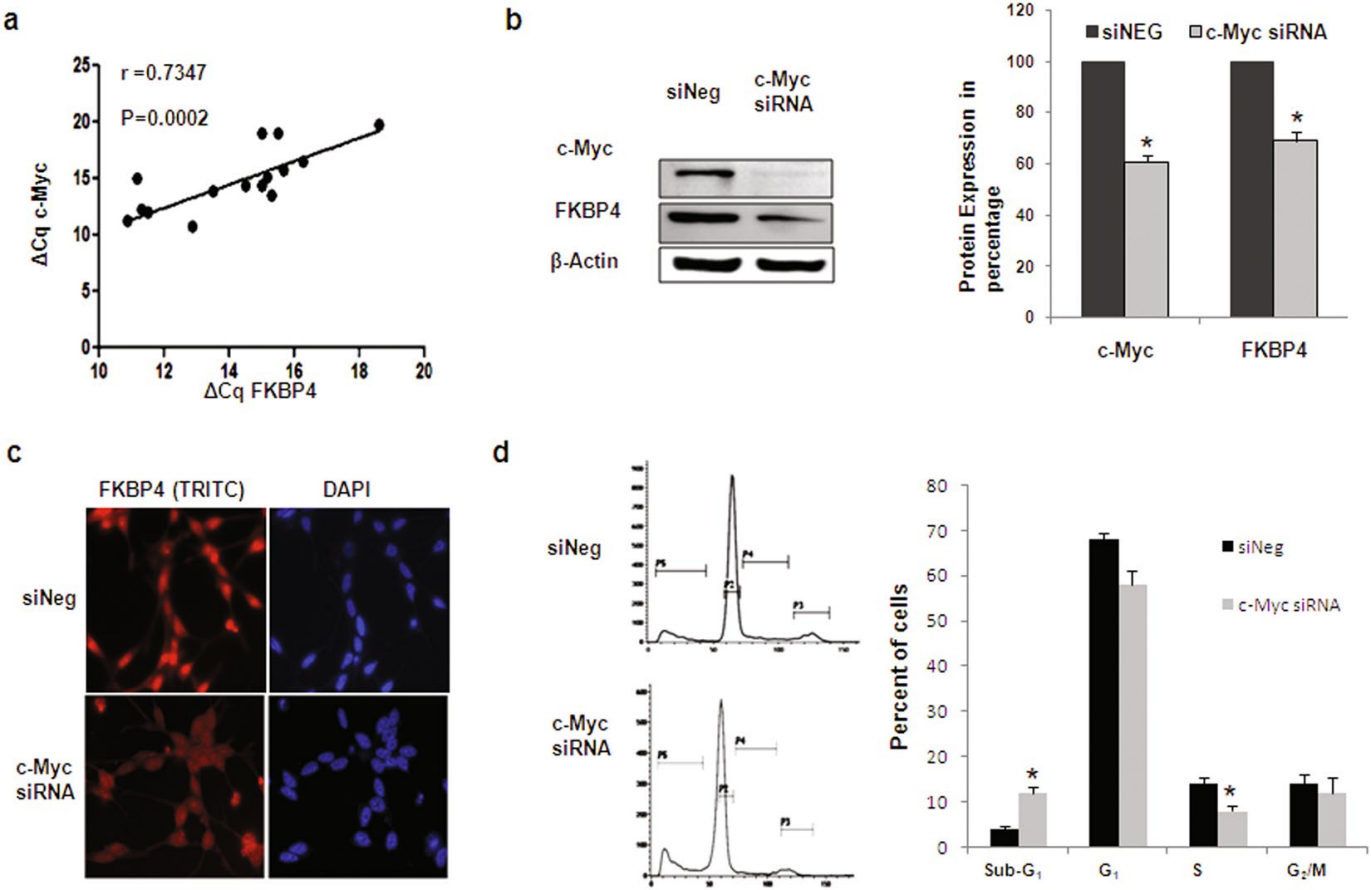
c-Myc regulated expression of FKBP4. (**a**) Scatter plot showing the correlation of gene expressions of c-Myc and FKBP4 in terms of ΔCq values by qRT-PCR. 18 S rRNA gene was used as an endogenous control. Pearson’s correlation coefficient (r) and P value were indicated in the plot. (**b**) Representative western blots and densitometric analysis of c-Myc and FKBP52 proteins following treatment of LNCaP cells with c–Myc specific siRNA showed significant downregulation in prostate cancer cell line (P < 0.05). β-Actin was used as a loading control. (**c**) Immunofluorescence images of LNCaP cells showing the expression of FKBP52 protein after transfection assay. The intensity of FKBP52 decreased after siRNA treatment. (**d**) Proportion of LNCaP cells in each phase of cell cycle following siRNA treatments. Bar diagrams represented the change in percent of LNCaP cells in different phases of cell cycle following siRNA interventions. (*) indicated the P value < 0.05.

*c-Myc* oncogene is commonly upregulated in prostate cancer. We observed that *FKBP4* expression was positively correlated with that of c-Myc in same set of tissue samples (r = 0.7347, p = 0.0002) (Fig. 4a). To investigate the functional implication of this statistical correlation, we examined the specific effect of c-Myc on FKBP52 expression by knocking down c-Myc expression in LNCaP. FKBP52 protein expression was significantly down-regulated (30%) after treatment with *c-Myc* siRNA for 48 hours, as supported by western blot analysis and immunofluorescence studies (Fig. 4b and c). Cell cycle analysis following *c-Myc* silencing showed reduced number of cells in the S phase and increased number of cells in the sub-G_1_ phase of the cell cycle by flow cytometry, indicating inhibition cell proliferation as a result of *c-Myc* siRNA transfection (Fig. 4d).

**Figure 5:**
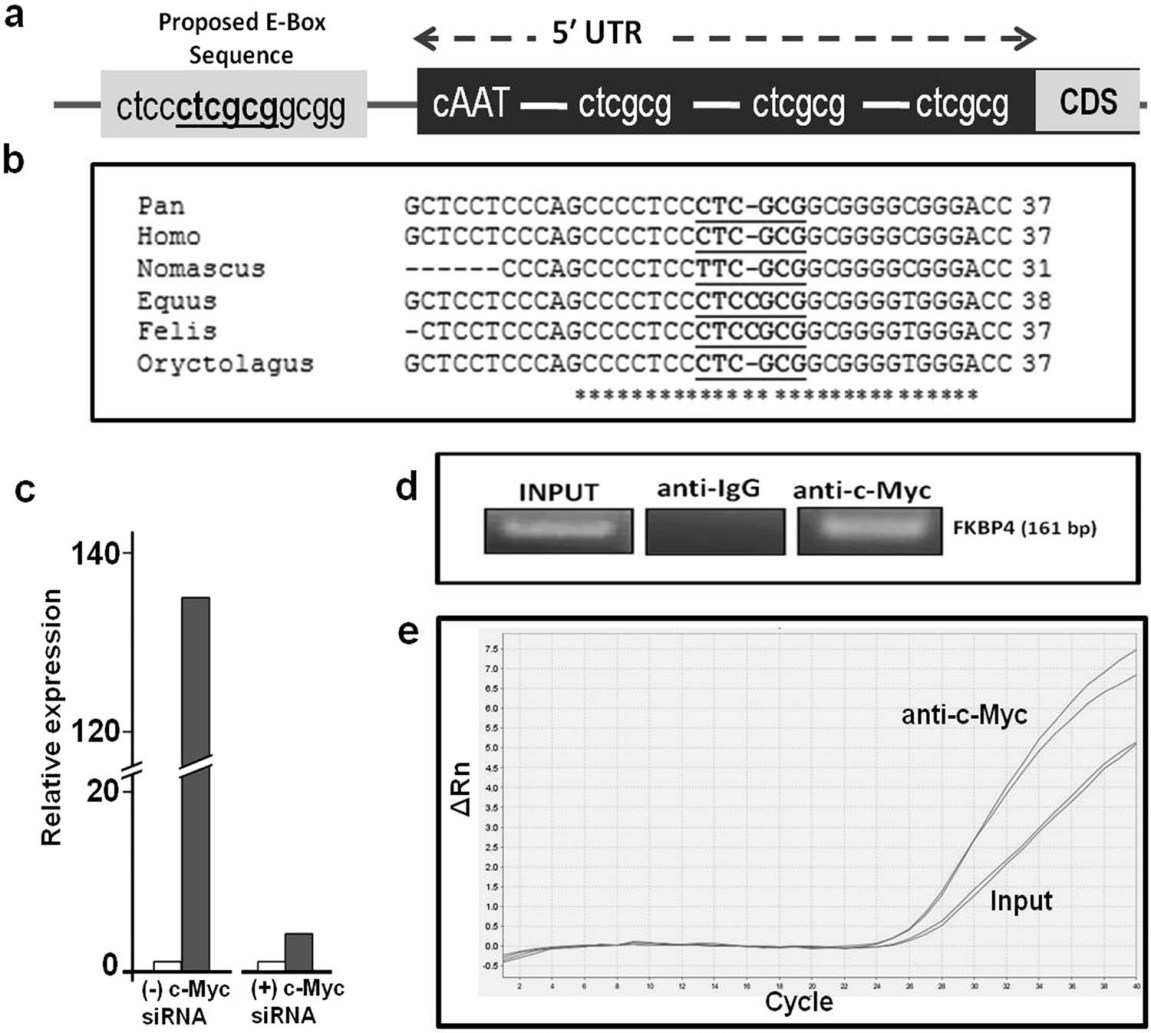
c-Myc binds to and regulates the FKBP4 promoter. (**a**) Map of the putative c-Myc binding sites on the FKBP4 promoter, 1 KB upstream of the translation start site. (**b**) Multiple sequence alignment of FKBP4 promoter sequences of taxonomically different organisms showing the most conserved site. (**c**) Bar diagrams representing the relative luciferase expression in FKBP4 construct with respect to empty pGL3 basic vector, in the presence or absence of c-Myc siRNA. (**d**) c-Myc antibody mediated chromatin immunoprecipitation coupled with PCR of genomic region flanking the most conserved E-box in the FKBP4 promoter, as represented by semi-quantitative RT-PCR. IgG was used as a negative control. (**e**) qPCR of FKBP4 promoter using INPUT and c-Myc specific ChIP samples.

A bioinformatic analysis using a promoter search tool (TFSearch) was conducted on the 1 kb genomic region upstream to *c-Myc* translational start site. Of the four putative non-canonical MYC-MAX binding E-Boxes located in the promoter region of the *FKBP4* gene, three were detected in the 5′UTR while the fourth was mapped to upstream of the 5′ UTR of *c-Myc* gene^19^ (Fig. 5a). This E-box sequence (CTCGCG) was found to be conserved among distant taxonomic organisms as shown in homology determination using multiple sequence alignment of these promoter sequences of different taxonomic groups with ClustalW (Fig. 5b). A 393 bp fragment corresponding to the 307 bp upstream and 86 bp downstream regions of 5′UTR of the *FKBP4* gene containing the conserved E-box sequence was cloned into a vector containing a luciferase reporter (pGL3 basic). A significant increase (2.5-fold) in luciferase activity was observed in the prostate cancer cell line LNCaP, transfected with the *FKBP4* promoter-luciferase vector compared to cells transfected with the empty reporter vector (Fig. 5c). A remarkable decline (32.89 fold) in the luciferase activity was recorded when the LNCaP cell were cotransfected with *c-Myc* specific siRNA compared to siNeg treated cells (Fig. 5c). In order to confirm the binding of c-Myc with the conserved E-box located in the *FKBP4* promoter, a chromatin immunoprecipitation (ChIP) assay was performed. PCR amplification of chromatin specifically complexed with anti *c-Myc* antibody using primers that covered the conserved *c-Myc* binding site in the *FKBP4* promoter confirmed recruitment of c-Myc in the promoter region of the *FKBP4* gene (Fig. 5d).

### FGF8 expression is modulated by c-Myc

**Figure 6:**
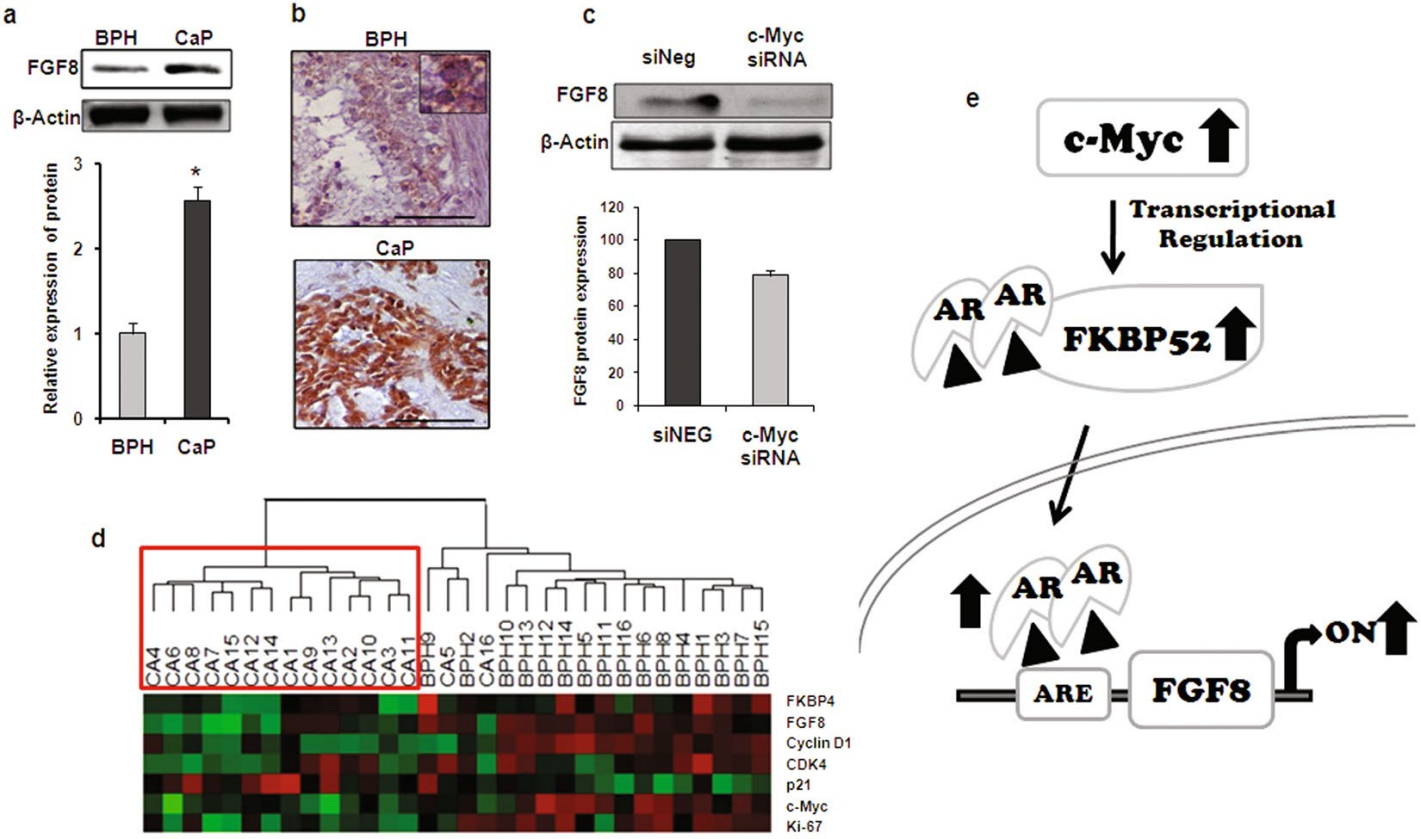
Silencing c-Myc down regulates FGF8 expression in prostate cancer cells. (**a**) Western blots showing the relative expression FGF8 protein in representative CaP and BPH samples. β-Actin expression was used for normalization. Bar diagram in the lower panel indicates the relative increase of FGF8 in cancer (n = 6) samples compared to that of BPH (n = 6) samples. (**b**) Representative immunohistograms of FGF8 expression in BPH and cancer samples. Scale bar: 50 µm. (**c**) Western blot analysis showing downregulation of FGF8 protein in the c-Myc siRNA transfected LNCaP cells. Bar diagram in the lower panel represents the percent reduction of FGF8 expression after transfection assay. (**d**) Heatmap showing two distinct clusters comprising BPH and cancer samples based on relative expression of genes, namely FKBP4, FGF8, cyclinD1, CDK4, p21, c-Myc and Ki67. Red boxes show higher ΔCt values, hence lower expression and green boxes show lower ΔCt values for higher expression in the corresponding samples. 18 S was used as an endogenous control. (**e**) A proposed model depicting the novel functional axis between c-Myc, AR and FGF8 operating through FKBP52 during prostate cancer progression. In prostate tumor, c-Myc expression is amplified. Upregulated c-Myc promotes FKBP4 gene expression, which in turn facilitates AR translocation to the nucleus. This is followed by activation of AR regulated genes, FGF8 being one of them.

FGF8 is known to house an androgen responsive element (ARE) in its promoter and is positively regulated by androgen levels in the blood^20^. Several of the biological processes that are enriched with differentially regulated genes from our microarray analysis were found to harbor *AR, FKBP4* and *FGF8* in same functional category (Table 1). The microarray analysis indicated that FGF8 was up regulated by 8.31-fold in prostate cancer tissues compared to BPH tissue which was further confirmed by western blot analysis (2.56 fold over-expressed in cancer tissues, p = 0.001) and immunohistochemistry in the study samples (Fig. 6a and b). Since the normalized Cq estimates of *FGF8* statistically correlated with those of *c-Myc* (r = 0.7139 and P = 0.0002) and *FKBP4* (r = 0.7347 and P = 0.0002), the expression of FGF8 was additionally analyzed in LNCaP cells following c-Myc was knocked down. FGF8 expression declined significantly (20%, p < 0.01) due to c-Myc silencing in LNCaP cell line (Fig. 6c). Finally, heat map analysis showed that prostate cancer and BPH tissues may be distinguished on the basis of the expression of the genes such as *FKBP4*, *FGF8, c-Myc* in addition to *cyclinD1*, *CDK4*, *Ki-67* and *p21* (Fig. 6d). Collectively our data suggested that FKBP52, the AR co-chaperone, was regulated by c-Myc and therefore raised the possibility of opening up a new therapeutic strategy to manage AR dependent diseases.

## Discussion

In an attempt to bestow a theranostic perspective to the current trend of research in prostate cancer, a differential gene expression was applied to identify putative molecules which would set apart disease progression states and act as specific targets in cancer prognosis and therapy in future. A comparative gene expression analysis conducted between human prostate cancer and BPH tissues revealed that several genes from the AR signaling pathway were up regulated. Among these genes, *FKBP4* showed ~5-fold up regulation in cancer tissues. Consistent upregulation of *FKBP4* in prostate cancer was also shown in other studies^21^. Immunohistochemical screening of BPH and cancer samples in patients validated over expression of the *FKBP4* gene at the protein level. Localization of FKBP52 indicated the protein was predominantly present in the cytoplasm of LNCaP cells as assessed by both western blot analysis and confocal microscopy.

Androgens are critical regulators of prostate differentiation and function, and of prostate tumor growth and cancer cell survival^22^. It is well established that the effect of androgens is mediated via the AR through regulation of the expression of several genes^23^. Amongst the major contributors of AR activity is FKBP52, a macro immunophilin molecule. The hormonally-induced replacement of FKBP51, another immunophilin and AR co-chaperone, by FKBP52 is considered to be one of the critical steps in the maturation and mobilization of the receptor to the nucleus in which FKBP52 directly interacts with Hsp90 which is already bound to hormone receptor^24^. Studies have confirmed contribution of FKBP52 in protein folding, ligand binding, and nuclear localization of AR receptors, finally to the AR-induced prostate cell growth^2^. Therefore, the intricate role of *FKBP4* in regulating prostate growth may be anticipated because its increase at both the transcriptional and translational levels have been observed in prostate tumor tissues compared to BPH tissues. In agreement with our findings recent proteomics analysis of malignant and benign prostate tissues showed dysregulation of AR chaperones namely chaperons FKBP52 and HSPD1^25^. Loss of FKBP52 activity is known to lead to male infertility through selective abrogation of AR activity and male FKBP52 KO animals showed defective development of the prostate gland^26^.

The development of potent androgen synthesis inhibitors and AR antagonists has remained the mainstay strategy in managing prostate tumors. The disease often develops resistance to these therapeutics, while it continues to depend on the AR signaling axis^27^. This necessitates innovative therapeutic approaches which would weaken AR signaling without directly targeting the androgens or their receptors. Thus development of FKBP52-specific small molecule inhibitors may be predicted to be a highly targeted strategy in diseases that are dependent on a functional AR pathway. More specifically, FKBP52 proline-rich loop may be targeted in relation to regulate the activity of the protein in AR-dependent diseases^5^. The current study makes an attempt to strengthen the current understanding of prostate cancer biology with respect to AR-co-chaperones by critically dissecting its regulation and accentuating possible downstream effectors.

To consolidate our understanding of the regulation of *FKBP4* gene in prostate cancer cells, the trend of several genes which display prominent roles in various types of cancer including prostate cancer was investigated. The list included *cyclinD1, CDK4, p21, c-Myc* and *Ki-67*. The first prominent contender was *c-Myc*, a proto-oncogene whose expression was strongly correlated with that of FKBP52. Our results showed that FKBP52 and c-MYC proteins were highly expressed in LNCaP cell lines as reflected by western blot and immunofluorescence studies. Further investigations identified *FKBP4* as a c-MYC target on the basis of the presence of a non-canonical E-box motif in the promoter region of *FKBP4* gene which was found to be conserved (consensus sequence CACGTG) amongst distant taxonomic groups. Presence of a non-canonical MYC-MAX-like binding site which may mediate the induction of *FKBP4* mRNA in cells with constitutive c-Myc expression has been reported by others^19, 28^. Additionally, *FKBP4* has been shown to be a target of c-Myc gene^11, 29, 30^. However, till date, to the best of our knowledge, there is no report of the role of a probable *Myc-FKBP4* axis in prostate cancer. To address this issue, we evaluated whether silencing c-Myc would have direct effects on the expression of *FKBP4* in prostate cancer cells. The analyses of c-Myc regulation of the promoter activities of *FKBP4* in LNCaP cells demonstrated silencing c-Myc repressed *FKBP4* promoter activity. A potential role for c-Myc in the development of androgen-refractory disease is indicated by the high frequency (up to 70%) of c-Myc amplification in androgen-independent prostate cancer (AIPC)^31, 32^. Presumably, of the various factors which lead to c-MYC gene amplification in prostate cancer, increased expression and activity of AR can increase c-Myc expression, which is a direct downstream target of AR^33^. In addition, AR regulates c-Myc transcription directly in a ligand-independent manner^14^. Collectively, this study delineated a linear co-relation between AR, c-Myc and FKBP52 in prostate cancer.

In addition, several cell proliferation-related genes viz. *Ki-67*, *cyclin D1* and *CDK4* were over expressed in prostate cancer, concomitant with significant down regulation of *p21* in prostate cancer. It has been proposed that cyclin D1 serves as a feedback inhibitor of the AR^34–36^. With increased CDK4 expression and over expressed proliferation marker Ki-67, it was clear that the samples hold the general proliferative scheme. Previously, p21 was known to be up regulated in prostate cancers since androgen up regulates p21 expression, suggesting that p21 may have an anti-apoptotic function in prostate cancer^37–40^. Our findings directly oppose the previous reports, and one possible explanation may be that c-Myc over expression inhibits p21 expression by interacting with Sp1/Sp3 transcription factors through the −119 bp to + 16 bp region of p21 gene that contains multiple Sp1 binding sites^41, 42^.

As revealed by the microarray analysis, the second prominent contender involved in prostate carcinogenesis was fibroblast growth factor 8 (*FGF8*). FGFs are polypeptide growth factors involved in various normal and pathologic processes. Elevated levels of various FGF ligands as well as aberrant expression of FGFRs have been reported in human prostate cancer and metastases^20^. It is well established that prostate carcinogenesis is adversely modulated by the presence of FGF8, and metastatic and more advanced AIPCs are associated with over expression of FGF8^43^. ChIP-based studies confirmed the presence of an ARE in the promoter region of *FGF8* ^20^. FGF8 is also involved in later stages of tumor progression by increasing tumor cell invasion and migration as well as angiogenesis and bone metastasis^44^. Based on the microarray data, our results confirmed that relative expression of FKBP4, c-myc and FGF8 were significantly higher in both low (Gleason score = 6) and high grades (Gleason score > 6) of cancer as compared to that of the mean BPH samples in a larger cohort. Since there is no report till date which may indicate a direct correlation between FKBP52 and FGF8, we investigated the probable effect of c-Myc silencing on the expression of FGF8 gene. Our results convincingly demonstrated that application of c-Myc siRNA abrogated the expression of FGF8 in addition to FKBP52. Thus, FGF8 could also be functionally connected to the already observed axis of AR, c-Myc and FKBP52 (Fig. 6e). The findings that FGF8 is elevated in human premalignant PIN lesions, suggests that this factor is involved in the early steps of prostate tumorigenesis in addition to its role in later stages of tumor progression by increasing tumor cell invasion and migration as well as angiogenesis and bone metastasis^46^. All these pieces of information corroborate with our results. Since FKBP52 is a strong agonist of the AR pathway together with other major players such as c-Myc and FGF8, a possible strategy for intervention of this observed circuitry emerges from the present study. Modulation of this co-chaperone may therefore hold theranostic significance for management and treatment of patients with prostate cancer.

## Materials and Methods

All methods were adopted from published protocols or instructions of the manufacturer, and were performed in accordance with the relevant guidelines and regulations.

### Materials

TRI^®^ reagent was purchased from Sigma Aldrich; High-Capacity cDNA Reverse Transcription Kit, Power SYBR Green Supermix and TaqMan^®^ Gene Expression Master Mix from Applied Biosystems; antibodies were purchased from Santa Cruz Biotechnology, USA; ECL reagent (SuperSignal^®^ West Pico Chemiluminescent Substrate) was procured from Thermo Scientific, USA; rabbit anti-cMyc antibody was purchased from Cell Signaling Technology; tissue culture media, serum and additional reagents were procured from HIMEDIA; RNase and cloning kits were purchased from Fermentas; HiPerfect transfection and silencing reagents and QIAprep^®^ Spin Miniprep kit were procured from Qiagen. Restriction enzymes were purchased from Thermo Scientific, USA.

### Sample collection and ethics statement

Tissue samples were collected from treatment-naive patients with BPH and CaP who underwent transurethral resection of prostate (TURP) or needle biopsy from the Saroj Gupta Cancer Centre and Research Institute (SGCC&RI), Microlap and Ramkrishna Mission Seva Pratisthan, Kolkata, under respective Institutional Review Boards with informed consent from patients. Information on demographic and clinical features was obtained through personal interview using a standard clinical questionnaire. The study was duly approved by the Biosafety and Human Ethics Committee, University of Calcutta.

All the patients who participated in the study were newly reported cases. The patients were examined by a panel of expert urologists and evaluated according to standard imaging procedures and laboratory analyses for benign hyperplasia or prostate cancer. Patients with prostatitis and high-grade prostatic intraepithelial neoplasia were excluded. Information on demography, family history and clinical parameters such as serum PSA, blood sugar, past infections and biopsy report confirming the malignancy were collected from all study participants. Gleason score of the biopsy samples and staging of tumors from the prostate cancer patients were ascertained by a panel of qualified pathologists at SGCC&RI.

### Study participants

The differential gene expression studies were carried between fifteen prostate adenocarcinomas and fifteen age matched BPH patients. The cancer samples used in this study consisted of 5 cases each of primary adenocarcinomas from both Gleason score 6 and 7, 4 cases from Gleason grade 8 and 1 from Gleason grade 9, respectively. The average age of the patients was 74.73 ± 9.39 with average PSA levels of 62.27 ± 3.56 ng/ml. BPH samples were taken from patients with an average age of 67.47 ± 7.60 and average serum PSA levels of 3.368 ± 2.02 ng/ml.

### Histopathologic and morphometric analysis

Freshly obtained TURP and biopsy tissue samples were either fixed in Bouins’ fluid for histological analyses by hematoxylin-eosin double staining method or snap frozen in liquid nitrogen and stored at −80 °C for RNA and protein extraction^46^. Tumors showing prostate weight ≥ 60 g and BPH nodules were selected as severe BPH. Low grade cancers were associated with small, closely packed glands resembling normal prostate tissue and considered as moderately differentiated (Gleason Score < 7). Tumors with loose glandular architecture and poorly differentiated tissue or often just sheets of cells throughout the surrounding tissue was diagnosed as high grade group or advanced stage of adenocarcinoma (Gleason Scores ≥ 7).

### RNA extraction and cDNA synthesis

Both low-grade and high-grade adenocarcinomas were selected for mRNA isolation. Other malignancies and known prostatitis cases were excluded. Tissue RNA was extracted by TRI^®^ reagent according to the manufacturer’s protocol and stored at −80 °C. cDNA was synthesized from 1 μg of total RNA using random primers and qRT-PCR was carried out in a GeneAmp® PCR System 9700 Thermal Cycler (Applied Biosystems). Total RNA was extracted from LNCaP cells with TRI^®^ Reagent. Phase separation was achieved by adding chloroform and centrifuging at 13000 rpm for 20 minutes at 4 °C. The aqueous layer was transferred to a clean tube and RNA was precipitated with isopropanol. The pellet was air dried and dissolved in DEPC-treated water. RNA was quantified, its quality checked by gel electrophoresis and stored at −80 °C for further use.

### TaqMan Assay

For the present study, ‘complete pooling’ method was applied. Pooled samples were created by adding an equivalent amount of total RNA from each individual BPH or CaP sample. Two different pools were created for BPH and CaP samples. To each well of the TaqMan^®^ Array plate, a cocktail containing TaqMan^®^ Gene Expression Master Mix and cDNA representing the benign or malignant tissue was added. About 100ng cDNA was used per 20 µl reaction for a single well. Array plates were run in 7900HT according to manufacturer’s protocol. Standard 96-well format TaqMan^®^ array plate containing the immobilized sequence-specific primers and TaqMan^®^ MGB probe (6-FAM™ dye-labeled) for ninety four genes belonging to the AR signaling pathway were used for the expression analysis. BPH tissue was used as a calibrator, while the adenocarcinoma as target. Two housekeeping genes, 18 S rRNA and GAPDH, were used as references for normalization. Only the genes with reproducible amplification curves were considered.

### qRT-PCR

Quantitative RT-PCRs were performed for 15 adenocarcinoma and 15 BPH samples in 7900 HT (Applied Biosystems) instrument, using Power SYBR Green Supermix and 1 μl of the 10X diluted cDNA template in a total reaction volume of 10 μl. The quantitative PCR for each sample was done in triplicate. Primer sequence, cycling condition and PCR product size for target genes is summarized in Supplementary Table 1. Negative controls without template were included in each set of PCR assays. The expression analysis study was based upon the Relative Quantification method which determines the changes in steady-state mRNA levels of a gene across multiple samples and expresses it relative to the levels of an internal control RNA. Since relative quantification was the goal, ΔΔCq model was used for the study. 18 S rRNA gene expression served as the endogenous control and co-amplified in the same PCR condition.

### Western blot analysis

The tissue samples were lyzed in ice-cold RIPA Buffer (150 mM NaCl, 50 mm TRIS, 0.1% TritonX-100 and 0.1% SDS containing protease inhibitors 4-2-aminoethyl benzenesulphonyl fluoride, EDTA, aprotinin, leupeptin and bestatin, SIGMA). The homogenate was centrifuged at 14000 rpm at 4 °C for 20 minutes and supernatant containing protein was collected. The concentration of total protein was determined by Bradford assay. For cells in culture, media was aspirated from the culture flasks. After washing with PBS, cells were scraped and centrifuged at 2500 rpm for 3 minutes to precipitate the cells. Supernatant was aspirated and ice-cold RIPA buffer was added to the tube for protein isolation as for tissue samples. 30–40 μg of proteins were fractionated by SDS-PAGE, electrically transferred to PVDF membranes and blocked with 5% bovine serum albumin in TBST (0.1% Tween 20 in TRIS Buffer Saline). Blots were subsequently incubated with primary and secondary antibodies, and developed using the ECL reagent^46^. β-actin was used as loading control. Experiments were performed in triplicates and patient samples were checked in random pairs of BPH and prostate cancer.

### Immunohistochemistry

Paraffin-embedded tissues sections (5 μm) were deparaffinized with xylene and rehydrated. Antigen retrieval was performed by boiling sections in 100 mM citrate buffer (pH 6.0).To block nonspecific binding the sections were incubated in BSA in PBS for 1 hour at room temperature. Endogenous peroxidase was blocked by incubation for 30 min with a solution of hydrogen peroxidase in methanol. Primary antibody (1:50) was added and incubated overnight at 4 °C in a moist chamber. After washing, sections were incubated with corresponding secondary antibodies (1:200) for 2 hours at room temperature. Peroxidase activity was visualized with 3, 3′-diaminobenzidine (DAB). The sections were lightly counterstained with hematoxylin, dehydrated through ascending grades of ethanol, xylene and finally mounted in DPX^46^.

### Cell culture

LNCaP, human AR responsive prostatic carcinoma cell line, and PC3, an AR independent prostate cancer cell line were purchased from NCCS, Pune. Cells were cultured in RPMI-1640 (HIMEDIA) media was used with 10% heat inactivated new born calf serum (HIMEDIA) and 1% antibiotic-antimycotic solution and incubated at 37 °C in 5% CO_2_ for 3 to 4 days. Media was changed every alternative day. Cells were passaged regularly when they reached 80–85% confluency. Cells were detached by standard trypsinization with Trypsin-EDTA solution^47^.

### Cell cycle analysis

Floating and adherent cells were collected by centrifugation and washed with ice-cold PBS. The cells were resuspended in 500 µl ice cold PBS and fixed with 70% (v/v) cold ethanol. Cells were washed in PBS, suspended in 500 µl PI/Triton X-100 staining solution with RNase A and incubated for 30 minutes at room temperature before analysis was done. Cell debris was removed by filtration through 60mm nylon mesh. Nuclear-emitted fluorescence was measured with a BD FACSVerse^TM^ (BD Biosciences). 10,000 events were analyzed from each run^47^. The percentages of cells distributed in the G_1_, S, G_2_/M and sub-G_1_ phases of the cell cycle were determined by analysis with the BD FACSuite™ software, version 1.0.5.

### Cytoplasmic and nuclear protein fraction separation from cell lines

Cytoplasmic and nuclear protein fractions were separated from cell lines using the hypotonic solution (15 mM NaCl, 2 mM MgCl_2_, 20 mM TRIS pH 7.5, 1 mM EDTA, and protease inhibitor). Cells were scraped in PBS and then centrifuged at 2500 rpm for 3 minutes. 200 μl of hypotonic solution was added to the pellet and incubated for 10 minutes after mixing. Cells were then homogenized in a Dounce Homogenizer and checked by Trypan Blue to ensure lysis. Lyzed cells were then centrifuged at 4500 g for 15 minutes at 4 °C. Cytosolic protein fraction was collected from the supernatant and 150 μl of hypotonic solution was added to the pellet containing nuclei and sonicated^48^. The solution was centrifuged at 13000 rpm to remove the cell debris and supernatant was collected as nuclei protein fraction. Proteins were stored at −80 °C for future use.

### Immunofluorescence analyses

Cells grown on glass cover slips were fixed with 4% paraformaldehyde (in PBS, pH 7.4). Cells were then incubated with 1% BSA in PBS-containing 0.25% Triton X-100 for permeabilization. Cells were subsequently incubated with 1% BSA in PBST, primary and secondary antibodies in 1% BSA in PBST, and color developed using DAB. The cells were counterstained with hematoxylin, dehydrated in ethanol and mounted in DPX. For negative controls, cells were incubated in the absence of the primary antibody^47^. For localization and co-localization studies of proteins, immunofluorescence was performed on LNCaP cells grown on cover-slips. After fixation, permeabilization, blocking and primary antibody incubation, the cover-slips were incubated with fluorescent-tagged secondary antibodies. Finally, the cells were counterstained with DAPI to visualize nuclei before mounting. As an anti-fade, 0.1% N-propyl gallate was added before mounting.

### Small interfering RNA (siRNA) transfection

LNCaP cells were transfected with small interfering RNA (siRNA) targeting human c-*Myc* mRNA (Hs_MYC_7, Qiagen) using HiPerFect transfection reagent (Qiagen) according to the manufacturer’s instructions. AllStars Negative Control siRNA (Qiagen) was used as negative control.

### Reporter plasmid construct for promoter assay

The sequence of FKBP4 promoter was retrieved from Genecard (http://www.genecards.org/). The primers were designed to amplify the promoter sequence approximately 307 bp upstream and 86 bp downstream encompassing the MYC-MAX binding sites. The oligonucleotide sequences for promoter cloning were FP: 5′-GTGGTACCGGAGTGATGTCGGGAGATCG-3′ and RP: 5′-AGctcgagGAGGCTTGAGCACCTCTGCA-3′ and the product size was 399 bp. KpnI (5′-GGTAC^C-3′) and XhoI (5′-C^TCGAG-3′) restriction endonuclease recognition sites were incorporated in the oligonucleotides for directional cloning. The promoter sequence was initially cloned into TA vector pTZ57R/T (InsTAclone™ PCR Cloning Kit, Fermentas, K1214) followed by subcloning into pGL3 basic reporter vector digested with KpnI (ER0521) and XhoI (ER0691).The orientation of the recombinant vectors was checked through sequencing and double digestion with the respective restriction endonucleases. Plasmid DNA was isolated by miniprep kit according to manufacturer’s instructions. DNA concentration was measured by spectrophotometer and stored at −20 °C.

### Co-transfection of reporter plasmid construct, c-Myc siRNA and Luciferase assay

LnCaP cells were seeded (1 × 10^5^) 14–16 hrs before transfection in 6-well plates. Cells were transiently transfected with i) empty 0.4 µg pGL3-Basic vector, 0.04 µg pRL-TK (Promega, E2241), ii) 0.4 µg FKBP4 promoter construct, 0.04 µg pRL-TK, iii) empty 0.4 µg pGL3-Basic vector, 0.04 µg pRL-TK, 4 µg c-Myc siRNA, iv) 0.4 µg FKBP4 promoter construct, 0.04 µg pRL-TK, 4 µg c-Myc siRNA with the help of HiPerFect transfection reagent. Forty eight hours later, cells were lysed according to the Dual Luciferase Reporter Assay protocol (Promega, E1910) and Firefly and Renilla luciferase activities were measured in GLOMAX 20/20 Luminometer (Promega, Madison, WI). 20 μl of cell lysate was transferred into luminometer tubes containing 100 μl LAR. Firefly luciferase activity was measured first, and then Renilla luciferase activity was measured after the addition of 100 μl of Stop&Glo Reagent. Renilla luciferase activity was normalized with respect to Firefly luciferase activity. Total protein was estimated by the Bradford method. All transfection assays were done in triplicate.

### Chromatin Immunoprecipitation (ChIP) Assay

Transcription factors and DNA were cross-linked with HCHO at 37 °C for 10 minutes and quenched with 1 ml glycine. Cells were scraped, collected in PBS and lysed using 1 ml SDS buffer with 10 µl PMSF and protease inhibitor cocktail. Sonication was standardized at 15 pulses (30/30 seconds) to produce genomic DNA fragments of ~250 bp. Supernatant was collected and pre-cleared by adding protein A-agarose/salmon sperm DNA beads to the chromatin. 200 µl supernatant was stored to use as input. 10 µg of anti-cMyc antibody was added and incubated at 4 °C overnight. Anti-IgG antibody was used as the negative control. The solution was incubated with blocked beads for 3 hours at 4 °C. Beads were washed and Chelax-100 reaction was performed to collect ChIP-DNA. Collected DNA was purified using standard phenol-ethanol precipitation method. ChIP-DNA was amplified by primers designed for amplifying c-MYC promoter region (Forward Primer: 5′ TCCCGCCGTCTCTAGAAAGTTC 3′ and Reverse Primer 5′ AGGAGGCTTGAGCACCTCTGCA 3′). The amplified products were analyzed both semi-quantitatively by gel electrophoresis and qRT-PCR.

### Statistical analysis and bioinformatics

The TaqMan^®^ array plates were analyzed with RQ Manager Software (1.2 version) for automated data analysis. Gene expression values RQ (Relative Quantification = 2^−ΔΔCq^) were calculated with SDS 2.3 software (Applied Biosystems). The relative transcript level was quantified in terms of RQ (Relative Quantification = 2^−ΔΔCq^) value where ∆Cq expression of a cancer sample was normalized to BPH control and represented by bar diagram. The difference of expression was calculated by Student’s t-test in GraphPad software (www.graphpad.com). Pairwise Pearson’s correlation co-efficient (r) and P value (two-tailed) were calculated by the ΔCq values of genes using GraphPad Prism 5 (graphpad-prism.software.informer.com/5.0). Densitometric analyses of western band were quantified by ImageJ software (imagej.nih.gov/ij) and P value was determined by Student’s t-test in GraphPad software. A value of p < 0.05 was considered statistically significant. In transfection assays, column plots were used to represent relative expression of the proteins that was 100-[{(siNEG-siRNA Target gene)*100}/siNEG]. The percent reduction of protein expression was plotted by bar diagrams. Cell population after FACS analyses was represented by bar diagrams. The bar diagrams of the luciferase assay indicated the relative expression of pGL3-FKBP4 promoter construct in respect of pGL3-empty vector, either in the presence or in the absence of c-Myc siRNA.

### Data availability

This is to state that all data generated and analyzed are included in the manuscript, and are available from the corresponding author on reasonable request.

## Electronic supplementary material

Supplementary Information
