# Supplementary material for: Pathway-based expression profiling of benign prostatic hyperplasia and prostate cancer delineates an immunophilin molecule associated with cancer progression
Source: Sci Rep. 2017 Aug 29;7:9763. doi: 10.1038/s41598-017-10068-9 (PMC5575002; doi:10.1038/s41598-017-10068-9)

**Pathway-based expression profiling of benign prostatic hyperplasia and prostate cancer delineates an immunophilin molecule associated with cancer progression**

Ankur Bhowal, Subhadipa Majumder, Subarna Ghosh, Sanmitra Basu, Debrup Sen, Susanta Roychowdhury, Sanghamitra Sengupta, Urmi Chatterji


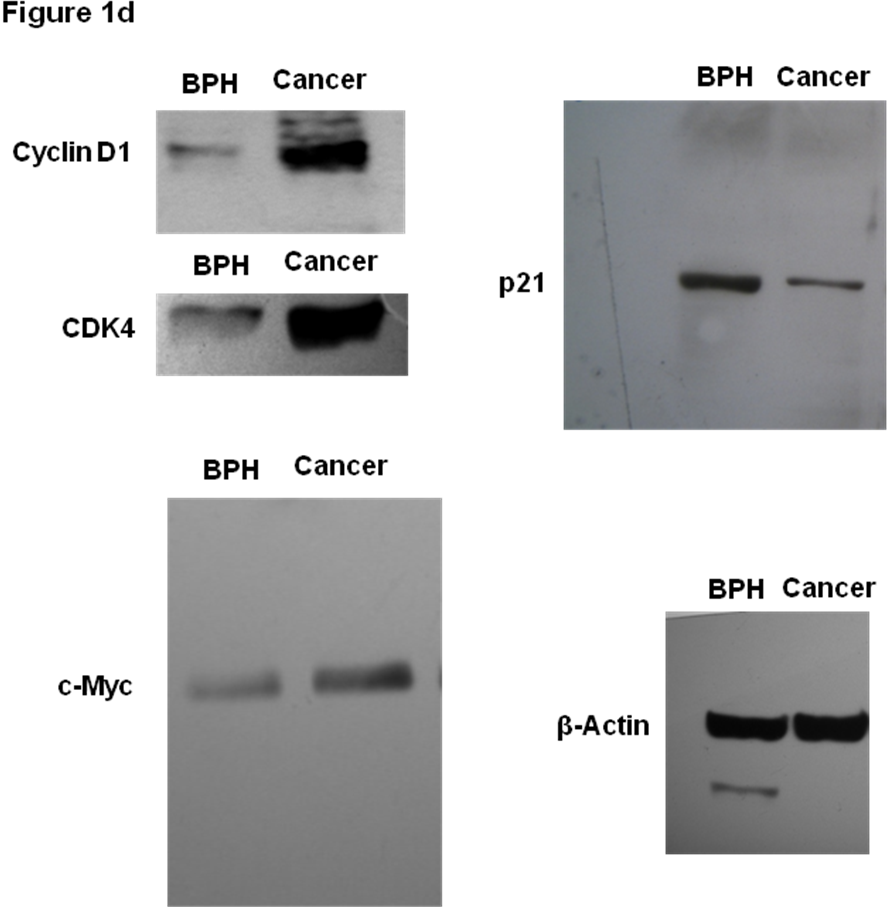


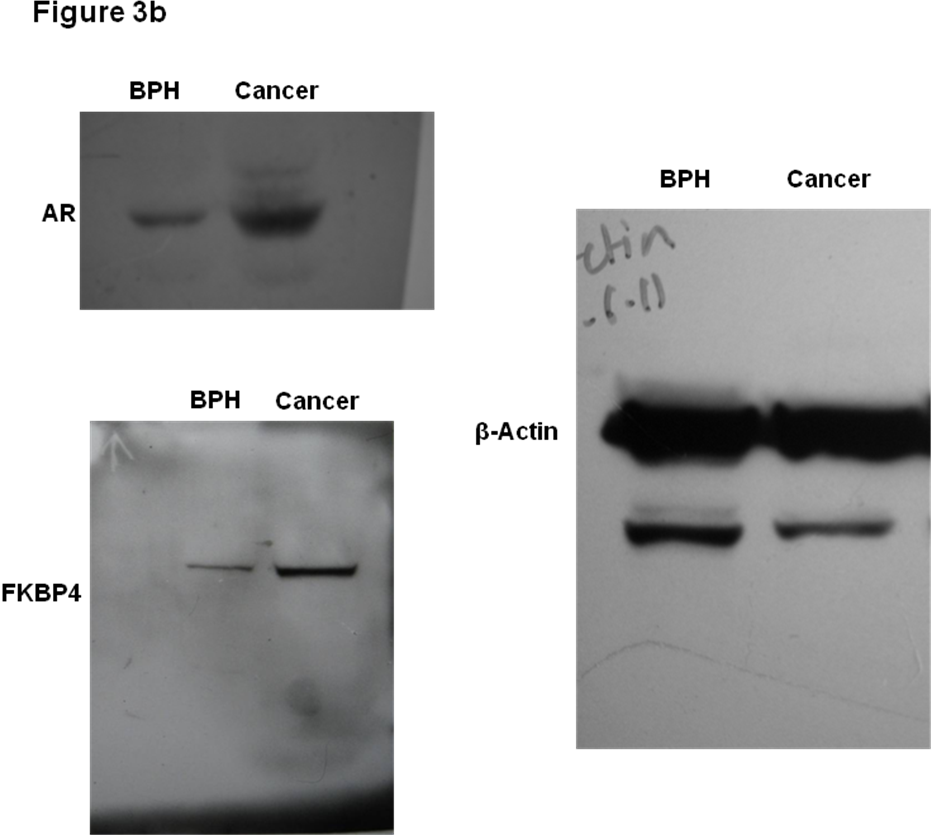


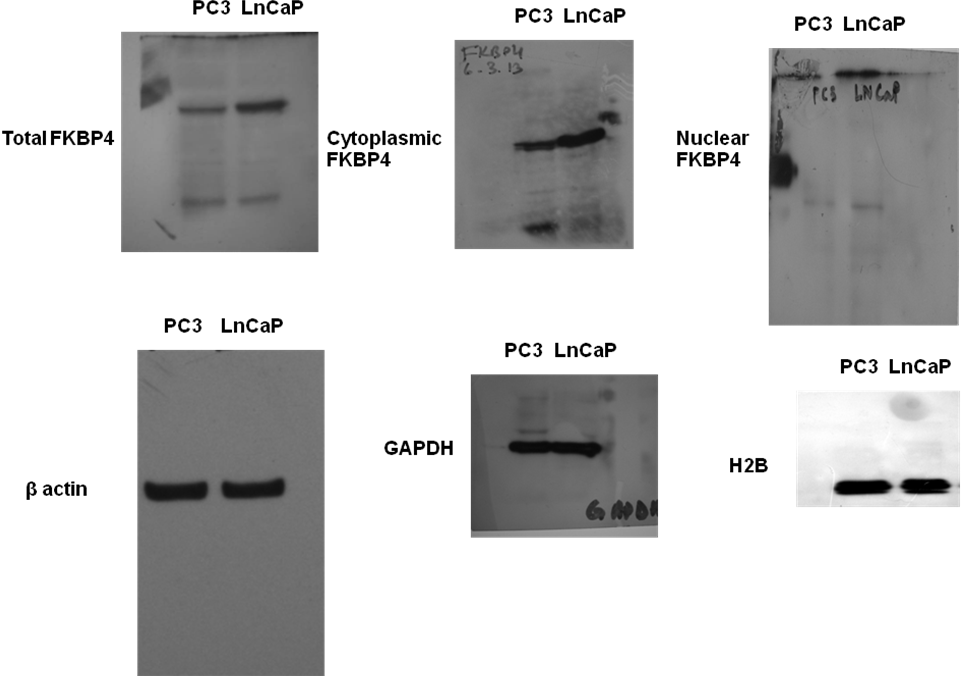


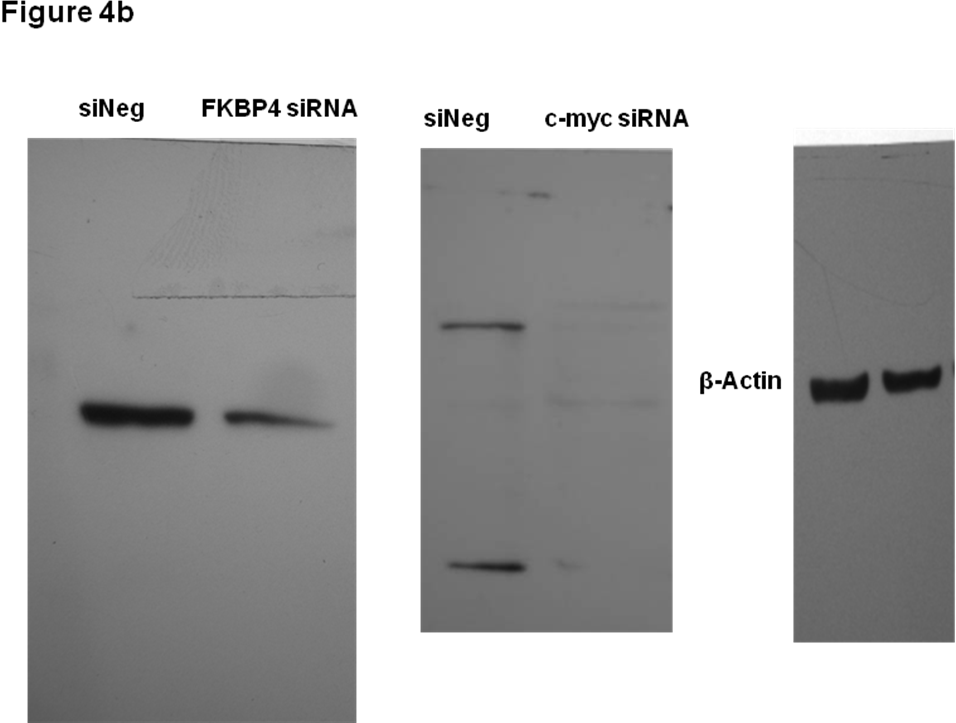


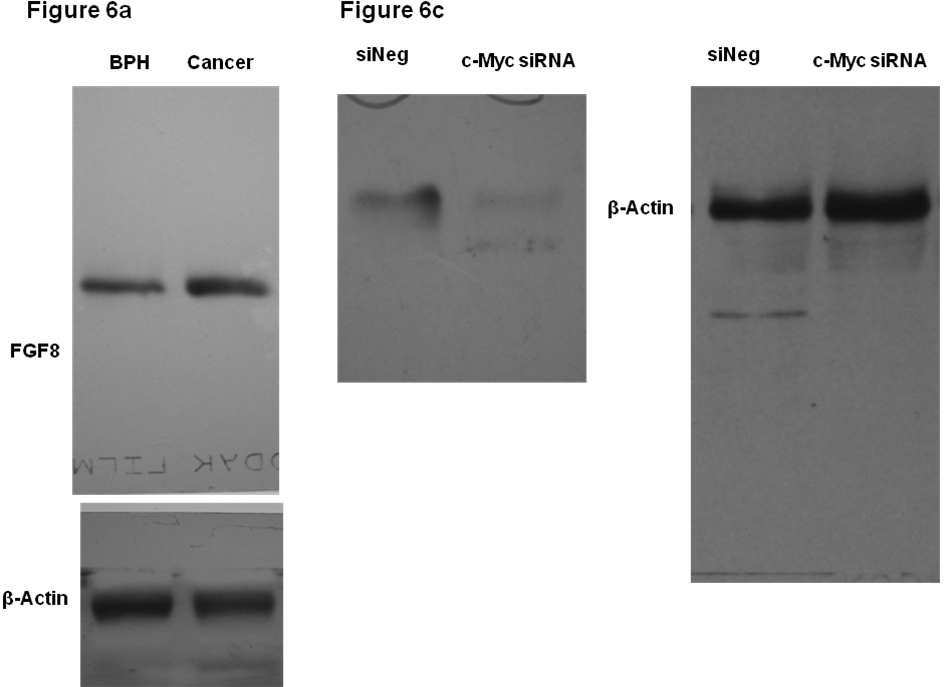

Supplement: Supplementary file 1 — Supplementary Information [file 41598_2017_10068_MOESM1_ESM.doc]
